# On the Feasibility of Low-Cost Wearable Sensors for Multi-Modal Biometric Verification

**DOI:** 10.3390/s18092782

**Published:** 2018-08-24

**Authors:** Jorge Blasco, Pedro Peris-Lopez

**Affiliations:** 1 Information Security Group, Royal Holloway, University of London, Egham TW20 0EX, UK; 2 Department of Computer Science, Universidad Carlos III de Madrid, 28911 Leganés, Madrid, Spain

**Keywords:** biometrics, verification, low-cost sensors, wearables, electrocardiogram, photoplethysmogram, accelerometer

## Abstract

Biometric systems designed on wearable technology have substantial differences from traditional biometric systems. Due to their wearable nature, they generally capture noisier signals and can only be trained with signals belonging to the device user (biometric verification). In this article, we assess the feasibility of using low-cost wearable sensors—photoplethysmogram (PPG), electrocardiogram (ECG), accelerometer (ACC), and galvanic skin response (GSR)—for biometric verification. We present a prototype, built with low-cost wearable sensors, that was used to capture data from 25 subjects while seated (at resting state), walking, and seated (after a gentle stroll). We used this data to evaluate how the different combinations of signals affected the biometric verification process. Our results showed that the low-cost sensors currently being embedded in many fitness bands and smart-watches can be combined to enable biometric verification. We report and compare the results obtained by all tested configurations. Our best configuration, which uses ECG, PPG and GSR, obtained 0.99 area under the curve and 0.02 equal error rate with only 60 s of training data. We have made our dataset public so that our work can be compared with proposals developed by other researchers.

## 1. Introduction

Authentication systems verify the identity of a machine or person to provide access to different services (banking, email, etc.). In general terms, an authentication system uses one or multiple factors, which can be categorised as “something you know” (i.e., passwords), “something you have” (i.e., security tokens), or “something you are” (i.e., biometrics). This is known as the authentication triad. Personal identification numbers (PINs) and passwords are routinely used to access computer systems, electronic locks, and all types of on-line accounts. Although they are probably the most widely used authentication factor, choosing good passwords is not a simple task [1], generally leading to weak choices, increasing the risk of compromise [2]. To avoid this, USB tokens and other hardware keys are gaining a broader adoption as a second-factor authentication method for greater security [3]. However, their main advantage is also their main weakness—they are physical objects that can be lost, with the attendant effect of disrupting access to services.

Biometric systems rely on physiological or behavioural characteristics that can be measured by a sensor and used to identify an individual. In contrast to passwords and security tokens, a biometric trait like a fingerprint or iris scan does not need to be remembered, and always goes with the user.

In the last few years, wearable devices have proliferated and found wide adoption among the general population. According to some estimates, wearable sales will rise to 100 million units by 2020 [4]. Equipped with an array of built-in sensors, wearables are very well-suited for biometric authentication [5]. By their nature, wearables are always with the user, which allows them to effectively perform authentication of the wearer [6]. In addition, wearers are not required to share their biometric traits with a third party, since all data can be stored inside the device. However, this nature also introduces new challenges. Because they are equipped with relatively cheap sensors, they capture noisier signals. In addition, due to their wearable nature, they can only be trained with data from the device user. These two issues hinder data collection in such a way that to-date, most proposals in the wearable area that use health-related signals have been developed relying on datasets captured with medical-grade equipment [7].

In this article, we make the following contributions: (i) We analyse the feasibility of using low-cost wearable sensors for biometric verification (i.e., training only with signals from the accepted user). Our analysis focuses on the electrocardiogram (ECG), photoplethysmogram (PPG), accelerometer (ACC), and galvanic skin response (GSR). We evaluate each signal on its own and in a multi-modal system under different scenarios and conditions. (ii) We make our dataset of wearable signals captured from 25 subjects public. This dataset will allow other researchers to develop and compare proposals under wearable conditions. (iii) By combining ECG, PGG, and GSR, we were able to build a verification biometric system with 0.02 equal error rate (EER) requiring only 60 s of training data. In contrast, when introducing the accelerometer, the EER increased to 0.11. We provide a more extensive analysis of our results in Section 4 and Section 5.

The rest of this article is structured as follows. Section 2 provides a background on biometrics. Section 3 explains our approach to wearable biometrics, explaining our feature extraction process and our matching algorithm. Section 4 describes our experiments and provides a summary of our results. Section 5 discusses the implications of our results and compares them to previous works. Finally, Section 6 gathers our conclusions and previews future lines of work.

## 2. Biometrics

Biometrics can be viewed as a pattern recognition problem in which a user who wants to be authenticated provides a set of physiological and/or behavioural characteristics to match a previously registered signature (or reference). Biometrics takes advantage of the fact that humans have natural diversity. A biometric system can be designed to perform identification or verification. When performing identification, the system tries to distinguish an individual from a population, whereas in verification it seeks to validate a person’s identity [8,9]. Wearables generally work in verification mode. As the subject is continuously wearing the device, they are intended to be set up and used with signals from their owner.

### 2.1. Biometric Verification Systems

**Figure 1:**
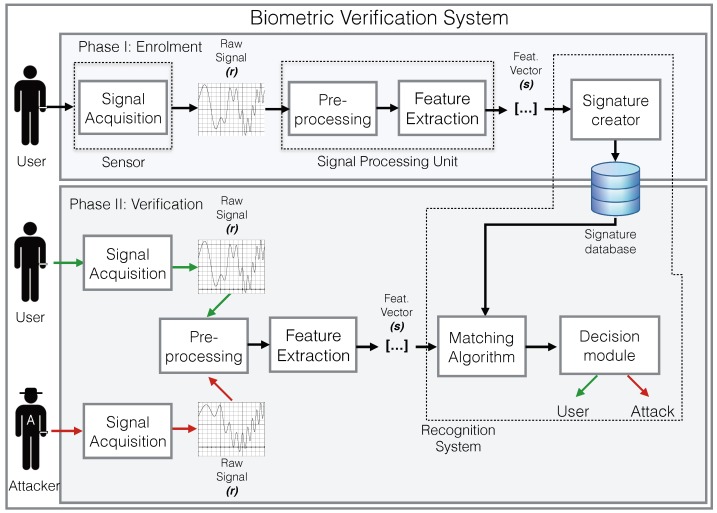
Functional components of a biometric verification system.

A biometric verification system is usually composed by the functional components shown in Figure 1: (i) a sensor (or set of sensors) that captures raw biometric signals (*r*); (ii) a signal processing unit that pre-processes and extracts feature vectors (*s*) from the signals (implements function P(r)→s); and (iii) a recognition system, which usually includes the signature (or template) database, and implements a matching algorithm B(s) [10].

During an enrolment or registration phase, the target user provides the reference signals to be used later during the matching process. In some cases, these are stored for later use. Alternatively, they are used for training a machine learning algorithm, and the model obtained after training (representing B(s)) is stored. Depending on the biometric signal and matching algorithm, the system may require several reference signals.

During the matching phase, the system is presented with a feature vector *s* that represents a subject’s raw biosignal *r* (or combination of signals), and the system must decide if *s* belongs to the target user *u* or not. This decision is based on the output of the pattern recognition function B(s). Generally, the output of B(s) is not binary (target or outlier), but a measure of the similarity of the vector *s* to previously stored vectors used as a reference. To calculate the decision, the output of B(s) is usually compared to a threshold θ. Thus, if B(s) is equal to or exceeds the threshold, then s∈u. Mathematically, this can be expressed as: s∈u if B(s)≥θ. It must be noted that B(s) is generally built using a set of *n* reference feature vectors $S^{u}=\left\{s_{1}^{u},s_{2}^{u},\cdots ,s_{n}^{u}\right\}$ belonging to the target user *u*. Although biometric verification systems can be presented with signals from different subjects (the registered one and the rest), they use only signals from the registered subject during the training phase. These systems are usually called one-class classification systems.

### 2.2. Biometric Signals

A biometric signal represents any physiological or behavioural characteristic that can be measured by a sensor and used to identify an individual. Not all physiological or behavioural measures are well-suited to biometrics. The requirements for a biosignal to be considered as a biometric may be different depending on the biometric system application. However, as a consensus among researchers, every signal should be universal, distinct, permanent, collectable, and inimitable to be considered for biometric authentication systems [11,12,13,14].

In this work, we evaluate the distinctness requirement of a set of signals captured from the wrist. We consider these signals to be universal and collectable, as anyone without a serious disability can wear a non-invasive wristband sensor to capture them. In this work, we do not directly address the long-term permanency of the signals. It has already been proved that some signals like the ECG slowly evolve over long periods of time [15]. This could reduce the overall quality of the system if the signature database is not updated after a long period of operation. However, we do address short-term permanency by comparing the results obtained while the subjects are performing different activities.

### 2.3. Common Pitfalls in Biometric System Evaluation

The performance, acceptance, and circumvention requirements of a biometric system are measured in terms of the errors the system makes when giving a decision. In biometrics, these are usually measured by the false positive rate (FPR), false negative rate (FNR), equal error rate (EER), and the area under the curve (AUC).

Unfortunately, many works only provide a subset of these metrics [16,17,18,19], which makes it very difficult to evaluate their quality. For instance, most of them report high accuracies, but those values only represent the number of times the system provides the correct answer and do not give any information about the system’s false positive or false negative rates. In fact, a system that classifies all samples as outliers could obtain a very high accuracy, if the sample set is unbalanced against outliers.

The EER reflects the point in the receiver operating characteristic (ROC) curve where the false positive and false negative rates are equal. Because of this, the EER is accepted as a good estimator of the quality of a biometric system. However, using the EER as a single quality metric can lead to two problems. First, it does not provide meaningful information about how the FPR and FNR may change when varying the threshold around that point (as a ROC curve does). This is of particular importance for biometric systems where one of them has to be prioritised over the other. Second, as described in [20], optimising this parameter without a proper analysis of the errors made by the classifier can lead to a classifier that provides a false sense of security. For example, if all false positives raised by a classifier are generated systematically by the same participant, all unauthorised access attempts carried out by that participant will go undetected. In this article, we provide and discuss our results for the common biometrics metrics (EER, AUC, FNR, and FPR). In addition, we also present and analyse the distribution of errors across our subject population and how that affects the overall security of the biometric system. We represent this with the Gini coefficient. This metric has already been proposed by [20], and offers a statistical measure of the dispersion of a distribution. In our case, we measure the dispersion of the distribution of errors across the participant population.

Although biometric systems can work either in verification or identification modes, many proposals based on wearable devices work in identification mode. This is unrealistic, as in most scenarios, the samples available during the enrolment (or system initialisation) are coming only from the device owner. This issue was also raised by [20] and, in fact, we were able to find only three other proposals that work in verification mode [16,17,18]. This fact, along with the difficulty of getting access to other works’ datasets, hinders the fair comparison of different proposals. To avoid these issues and foster fair comparison and evaluation of proposals, we are making our code and dataset freely available to other researchers (the dataset is available at: https://www.dropbox.com/s/lei4a27fcgp0ygr/LowCostSensorsBiometrics.zip?dl=0).

## 3. Low-Cost Sensors for Biometrics

In this section, we describe the design of our wearable biometric system. This includes, as described in Section 2: the raw signals captured by the biometric system, how our signal processing unit combines signals from different sensors to extract the feature vector, and how the matching unit executes the enrolment and verification phases.

### 3.1. Sensors

**Figure 2:**
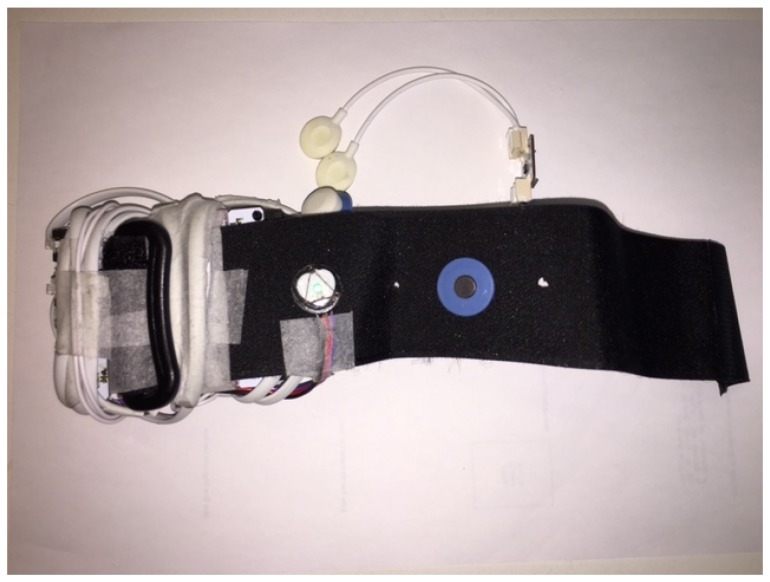
Low-cost hardware prototype for biometric verification.

The nature of the wearable scenario introduces some constraints on the sensors in the biometric system. Wearable sensors must be placed in a comfortable location that does not restrict the user’s everyday activities while capturing signals with the required quality for a biometric system. Our biometric system captures the PPG [21], ECG [22,23], ACC [16], and GSR [24,25] signals from the wrist. Our sensor selection is motivated by their availability in current fitness trackers and smart-watches, as well as their usability for other applications such as heart-rate measurement (in the case of ECG and PPG) and stress levels (in the case of GSR) [26]. Unfortunately, commercially available smart-watches do not provide access to raw data through their application programming interfaces (APIs). To avoid this restriction, we built our own hardware prototype to collect data from our experiments (see Figure 2). Our prototype is quite rudimentary, and it is not comfortable enough to wear over long periods of time. Nevertheless, the sensors used can be found in many fitness trackers and smart-watches currently being sold. We thus assume that using this kind of configuration does not interfere with most everyday activities while being able to capture signals without many artefacts.

Our prototype uses the ECG, ACC, and GSR sensors from Bitalino [27] and a low-cost open hardware PPG sensor [28]. For all the participants, the device was placed on their wrist, as in most fitness trackers. The PPG sensor was placed on the inner side of the wristband. One of the ECG electrodes was placed on the inner side of the wristband, while the second was placed on top of the wristband. The GSR electrodes were placed on the lower region of the palm of the hand. The accelerometer was attached to the microcontroller unit (MCU), also provided by Bitalino, where all sensors were connected. The MCU was subsequently connected to a Bluetooth block, allowing for real-time transmission of synchronised data to a smartphone.

#### 3.1.1. Photoplethysmogram

A photoplethysmogram, also known as PPG or pulse oximeter waveform, measures the change in blood volume during a cardiac cycle [21]. PPG sensors are composed of a light source and a photodetector. The light source is used to illuminate the tissue while the photodetector measures the variations in the intensity of absorbed or reflected light when blood volume varies. This sensor is based on the fact that the amount of light perceived by PPG photodetectors varies depending on the blood volume, blood vessel wall movement, and the orientation of red blood cells [29].

PPG sensors can be configured to detect light variations when transmitted through the skin (by placing a light emitter and photodetectors on opposite sides of a finger) or when the light is reflected from the skin (light emitter and photodetector are placed side-by-side). Transmission-based PPG sensors are difficult to use for daily activities, but reflection-based ones can be easily placed in contact with the skin on several parts of the body (wrist, ears, etc.).

PPG sensors have become very popular thanks to fitness trackers and smart-watches such as the Fitbit Charge HR [30], Apple Watch [31], and some earphone models that measure the heart rate from the ear [32]. PPG sensors are also available in the form of open hardware platforms [28], such as the one used in this work. Our PPG sensor measures the PPG signal from the wrist, like most fitness trackers.

One weakness of PPG sensors is that colour variations over the skin can be captured from a moderate distance using a video camera. In [33], the authors perform an in-depth analysis of this matter. As shown in this study, the success of the attack is conditioned to an advantageous and even quite unrealistic scenario—the individuals stay still and are observed under favourable illumination conditions. Even if an attacker was able to replicate these conditions and capture PPG traces, there is no known method to replay them to spoof a PPG sensor.

#### 3.1.2. Electrocardigram

**Figure 3:**
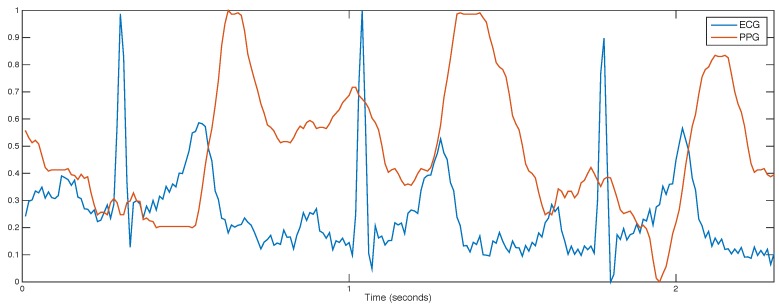
Synchronised photoplethysmogram (PPG) and electrocardiogram (ECG) signals collected from the wrist during 2–3 s. Signals were smoothed and normalised in the range 0–1.

An electrocardiogram (ECG) is a physiological signal that measures the electrical activity of the heart. An ECG signal generated from one heartbeat is composed of two intervals (PR and QR) and a U wave, which is usually invisible. The lengths of the different intervals and waves can be used to diagnose a wide range of medical conditions [22]. In Figure 3, an ECG and PPG traces of three heart beats are displayed.

An electrocardiogram (ECG) sensor consists of at least two metal electrodes that must be in direct contact with the skin. Medical ECG equipment uses three, five, or ten electrodes placed across the chest, wrists, and ankles, which is not suitable for wearable devices. Fitness chest straps and ECG t-shirts [34] use just two electrodes positioned across the chest to measure ECG and capture the heartbeat. Similarly, ECG sensors can be worn on the wrist [35]. In this work, we decided to obtain the ECG signal with a wrist wearable based on sensors provided by the Bitalino platform [27].

The existing ECG biometrics systems offer a high-security level against impersonation attacks. In one unlikely case, in 2017 Eberz et al. presented the first attack in which a synthesised ECG signal was successfully injected into an ECG wristband [23]. Although the novelty of the attack is undoubted, its consequences are limited since the authors assume that the ECG signal of an individual is captured via another ECG sensor and then a customised version of this signal is injected in the ECG band.

#### 3.1.3. Galvanic Skin Response

The galvanic skin response (GSR or skin conductance) measures the electrical conductance of the skin [24]. GSR sensors can be placed on any part of the body, but require direct contact with the skin. A GSR sensor is composed of two electrodes placed approximately one inch apart. This sensor sends a small, human imperceptible, amount of electrical current through one electrode and measures the intensity of the current received on the other.

The skin conductance varies depending on the amount of moisture (induced by sweat) in the skin. Sweating is controlled by the sympathetic part of the nervous system, so it cannot be directly controlled by the subject. The skin conductance can be used to determine body response to physical activity, stress, or pain. Previous research shows that the body response against these stimuli differs from person to person [36]. In this work, we capture the GSR signal with a wrist wearable based on sensors provided by the Bitalino platform [27].

**Figure 4:**
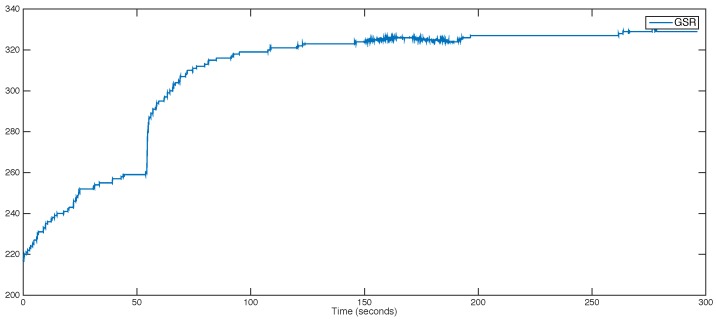
Evolution of the galvanic skin response of a subject over a period of 5 min.

GSR signals are widely used as a distinguisher of cheating conditions [25]. It is well-known that GSR values significantly increase when the subject is under stress conditions [37]. An attacker who has managed to capture the GSR values of a victim could try to impersonate them through modification of their GSR levels by forcing them to watch violent or unpleasant videos—alternatively, the attacker could overheat the room in order to cause the victim to sweat and thus cheat the GSR sensor. As an example, Figure 4 shows the evolution of the galvanic skin response of a subject during 5 min.

#### 3.1.4. Accelerometer

An accelerometer measures acceleration in *g*s (1 *g* is 9.81 m/s${}^{2}$) in the three axes: *x*, *y*, and *z*. Accelerometers are widely used in smartphones, fitness trackers, and other electronic devices. The most commonly known use cases are the detection of device orientation, step counting, and motion-based controls for games. The vast amount of data provided by accelerometers has proved useful in many other scenarios. When attached to the leg, hip, and hand, accelerometers have been successfully used as a replacement for video feeds in gait recognition [16]. Accelerometers in smartphones and smart-watches can also leak keystrokes, which could be used by malicious apps to extract PINs, passwords, and other sensitive information [38,39]. In addition, recent works have shown the potential use of gait as a complementary or full identifier in human recognition systems [40,41].

In our work, we use the accelerometer provided by the Bitalino platform [27]. As our main aim is to obtain gait data, we only require the acceleration on a vertical axis from the floor (*z*). This allows capturing the vertical forces that are generated by the pendular movement of the arms while the subject is walking.

To the best of our knowledge, the only known method to perform a sensor attack against an accelerometer-based gait recognition system is imitation attack. In these, the adversary mimics the gait of the victim after a period of direct observation. Muaaz and Mayrhofer proved that this kind of attack is highly unlikely, even when executed by skilled actors with similar physiological characteristics to their victims [42].

### 3.2. Signal Processing Unit

We applied the same preprocessing for cardiac (i.e., ECG and PPG) and accelerometer signals. First, the DC component is eliminated. Then, a band-pass filter is used, mainly to remove the power line noise. The lower and upper cut-off frequencies were set to 0.67 and 45 Hz, respectively. This removes the noise caused by the respiration of the subject (i.e., 0.67 Hz) and the power line noise (i.e., 45 Hz). We found the same filter to also be useful when removing noise from the ACC signal, as most of the information resides in the lower frequencies (20 Hz). Due to its simplicity, we only apply smoothing to the GSR signal.

We split each filtered signal into 2-s windows. This allows us to capture at least one full heartbeat per window, and is in line with window sizes for fiducial feature extraction [43]. The extraction of fiducial features (time domain) for the cardiac signals did not prove reliable due to signal noise. We use the Walsh–Hadamard (1) and Fourier (2) transforms to extract features from the cardiac and accelerometer signals. These transforms are especially suitable when dealing with cyclic signals (e.g., heart-based signals and gait) and can be extracted with a relatively low computational cost.

Assuming a raw signal, r(n), of length *N*, the discrete Fourier (*F*) and Walsh–Hadamard (*H*) transforms are mathematically described as follows:

(1) $$\begin{array}{cc} F[n]= & \sum_{k=0}^{N-1}r\left(n\right)e^{-j\frac{2\pi }{N}nk}\left(n=0:N-1\right), \end{array}$$

(2) $$\begin{array}{cc} H[n]= & \sum_{m=0}^{N-1}r\left(m\right)\prod_{i=0}^{N-1}{\left(-1\right)}^{m_{i}k_{i}}\left(n=0:N-1\right). \end{array}$$

Since most of the information resides in the lower frequencies, the 64 (Hadamard) and 32 (Fourier) lower coefficients were the values used in our experimentation (96 coefficients overall for each ECG, ACC, or PPG window). As the evolution of the GSR signal can be characterised by a small number of points, we only extract a set of statistical metrics (i.e., average value, standard deviation, maximum, and minimum) from each window. Table 1 summarises all features that can be extracted from the *j*th 2-s window. When used together, we can generate a feature vector of length 292.

**Figure 5:**
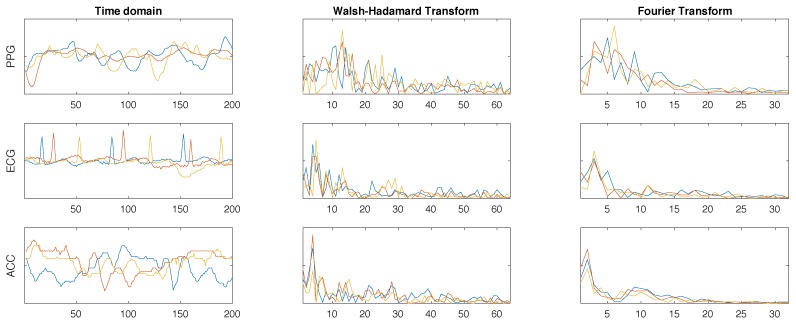
Three windows of PPG, ECG, and ACC signals from participant 3 in the time domain (after filtering) and their corresponding Walsh–Hadamard and Fourier transform coefficients.

Figure 5 shows three windows of signals taken from participant number 3 and their corresponding Walsh–Hadamard and Fourier coefficients used as features. The selected transforms highlight some similarities in the frequency domain that cannot be seen in the time domain due to the lack of synchronisation of the time windows.

### 3.3. Matching Unit

Our matching unit is a one-class classifierthat was designed by density estimation (boundary methods and reconstruction methods are two other alternatives to design this type of classifier [44]). Regarding the estimation of the training data density, three possibilities were considered: (1) Gaussian Model; (2) mixture of Gaussians; and (3) Parzen. We opted for the first option due to its simplicity and the good performance of the classifier, which is defined as follows:

(3) $$\begin{array}{c} B\left(s\right)=\left\{\begin{array}{cc} target & :{\left(s-\mu \right)}^{T}\sum^{-1}\left(s-\mu \right)\leq \theta ,\\ outlier & :{\left(s-\mu \right)}^{T}\sum^{-1}\left(s-\mu \right)>\theta , \end{array}\right. \end{array}\;$$

where μ and ∑ represent the mean and covariance matrix and are estimated using the sample values. The threshold θ represents the percentage of training target samples that will be rejected and classified as outliers. Note that the testing set might deviate from this behaviour since this is only optimised during training. The parameter θ was tuned through experimentation and finally set to 0.1 We trained one classifier per subject. During our experiments, we evaluated the impact of different training set sizes on the quality of the obtained classifiers. This is described in more detail in Section 4.

During testing, the remaining participant samples were considered as targets and samples from other participants were outliers. In our experiments, we evaluated the quality of the system depending on the number of 2-s windows used to make a decision. Instead of using a single sample to output a decision, a sliding window captured *n* samples which classification was combined to output the decision. In this case, and using the classifier defined in Equation (3), each sample $s_{j}$ is fed to the classifier B(·) and the output of all the windows is summed to generate the final classification:

(4) $$\begin{array}{c} B(s_{j}\left|\right|s_{j+1}\left|\right|s_{j+2}\left||\ldots |\right|s_{j+w_{size}})=\sum_{i=j}^{j+w_{size}}B\left(s_{i}\right). \end{array}\;$$

In our experiments, we evaluated how the different window sizes affected the classifier metrics. We also tested other strategies to combine the outcomes for each window (e.g., mean, median, or voting combiner), but they were discarded, as the addition offered the best results in terms of the studied performance metrics.

## 4. Experiments and Results

Our experiments were focused on evaluating the impact of the selection of signals, training set size, and verification window size on the quality metrics of the proposed classifier under four different scenarios.

### 4.1. Data Collection

We used the prototype to collect data from a group of 25 participants between 18 and 42 years old (with an average of 28.2 and median of 27, 16 males and 9 females) (the dataset is available at: https://www.dropbox.com/s/lei4a27fcgp0ygr/LowCostSensorsBiometrics.zip?dl=0). The data collection process was reviewed and approved by an ethics committee. Each participant was monitored by a member of the research team during the execution of each activity. Participants received a £5 gift card in return for their time, which was around 20 min. Each participant was asked to wear the prototype while performing the following activities in this particular order:

1. Sitting for 5 min while touching the second ECG electrode with a finger from their opposite hand. The ACC signal in this activity was discarded, as the participant did not perform any substantial movement.
2. Walking for 5 min through a corridor of approximately 15 m. This light exercise was introduced to capture the gait from the wearer and to introduce variations to the heart-based signals. During this activity, the participant was not required to touch the ECG electrode.
3. Sitting for 3 min (after the gentle stroll). During this final activity, the subject was asked to repeat the first activity, but for just 3 min. Again, the ACC signal was discarded.

The prototype was connected to an Android phone, which created a new recording session for each of the activities using a sampling frequency of 100 Hz. The sampling rate was also set to 100 Hz on the sensors used. Table 2 summarises the activities executed by each participant during the data collection process.

### 4.2. Scenarios

Our experiments were executed over four different scenarios to verify the suitability of the sensors (and their signals) across different situations. Our four scenarios, summarised in Table 3, were as follows:

- Scenario 1 used data from the same activity for training and validation. This is the most widely used experimental setting in biometrics works. To select training samples, we randomly chose a point between the first sample and |set|−|trainset|. Then, we picked all consecutive samples until |trainset|. This mimics real situations where the system is trained with successive samples [20]. We had two versions of this scenario: (1a) We used data from the first activity. We evaluated the different combinations of ECG, PPG, and GSR for their usage in biometric verification systems; and (1b) We used data from the second activity, evaluating ACC, PPG, and GSR. These scenarios could be combined to create a system that verifies the identity of the wearer in two different situations: sitting and walking.
- Scenario 2 evaluated the distinctiveness and short-permanency of the signals after a light exercise. We used samples from the first activity for training, but performed verification with samples from the third activity. We used ECG, PPG, and GSR signals from both activities and the same training set selection strategy from Scenario 1.
- Scenario 3 evaluated the distinctiveness property when the system was trained with more than one activity. This emulated a scenario where the user regularly updates the system with new samples. Both the training and validation were executed with activities one and three. The training set included random samples from both activities.

### 4.3. Experiments

We evaluated how using different *features*, the window size and the number of samples used to train the system affected the quality of the biometric system. For Scenarios 1a, 2, and 3, we executed experiments with [ECG], [PPG], [ECG, GSR], [PPG, GSR], [ECG, PPG], [ECG, PPG, GSR]. Scenario 1b replaced ECG with ACC, using the following sets: [ACC], [PPG], [ACC, GSR], [PPG, GSR], [ACC, PPG], [ACC, PPG, GSR]. We do not show the results for the GSR features, as they were by far the worst.

The number of training samples can have a significant impact on the classifier metrics. Using very few samples will result in a very general classifier that may produce many false positives. On the other hand, too many training samples may lead to overfitting and may affect the acceptability of the system, as the target user will have to spend a lot of time during the enrolment phase. We executed experiments where 10% (30 s), 20% (60 s), 30% (60 s), 40% (120 s), 50% (150 s), and 60% of samples (180 s) from the target user were used for training for Scenarios 1a, 1b, and 2 and 48, 96, 144, 192, 240, and 288 s, respectively, for Scenario 3.

The window size used during verification can improve metrics such as the false positive rate at the cost of increasing the response time. If the classifier makes very few mistakes with a single 2-s window, we can eliminate them by increasing the number of windows evaluated before making a decision. As described in Section 3.3, our matching unit sums the output of the classifier for the *n*-sized windows. In our experiments, *n* can be equal to 1, 3, 5, 8, and 10.

For each scenario, we executed one experiment for each of the possible combinations of parameters described in this section. All experiments were executed three times, averaging the results obtained in the three executions. Overall, we performed 25×4×6×6×5×3= 54,000 executions (subjects, scenarios, feature options, training sizes, window sizes, and repetitions, respectively).

### 4.4. Results

In this section, we show the results obtained grouped by scenario. For each scenario, we first analysed the equal error rate (EER) obtained by the different variations of the feature options, training, and window sizes (for the sake of completeness, the area under the curve (AUC) results for the three scenarios are summarized in Appendix A). We averaged the results obtained by all participants and repetitions. Then, we discuss how the best scenario configurations behaved regarding EER, distribution of errors, and false negatives and false positives, across the participant population.

#### 4.4.1. Scenario 1a

**Figure 6:**
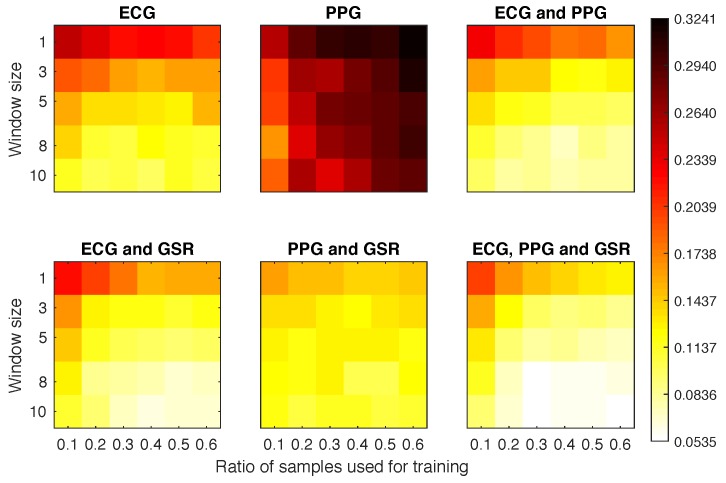
Equal error rate (EER) obtained for each experimental configuration in Scenario 1a.

The best EER obtained for experiments using 10% of the training samples (30 s) was 0.16, independent of the number of samples used for testing (Figure 6). The worst results were obtained when using the PPG signal on its own, except with bigger window sizes, when it could achieve an EER of 0.1653 (with window size 8 and 10% training set). This is probably because the captured PPG signals were subject to too much noise, hindering the ability of the system to find a specific pattern. With the ECG only, we obtained an EER below 0.11 with 10 as the window size and 30% of training data. When adding the GSR, we obtained the same results with smaller window and training sizes. If we combined all three signals we were able to obtain 0.09 EER with only 5 windows and 20% of the training set (60 s). If we increased the training set size to 90 s and the window size to 8 we were able to further reduce the EER to 0.057.

#### 4.4.2. Scenario 1b

**Figure 7:**
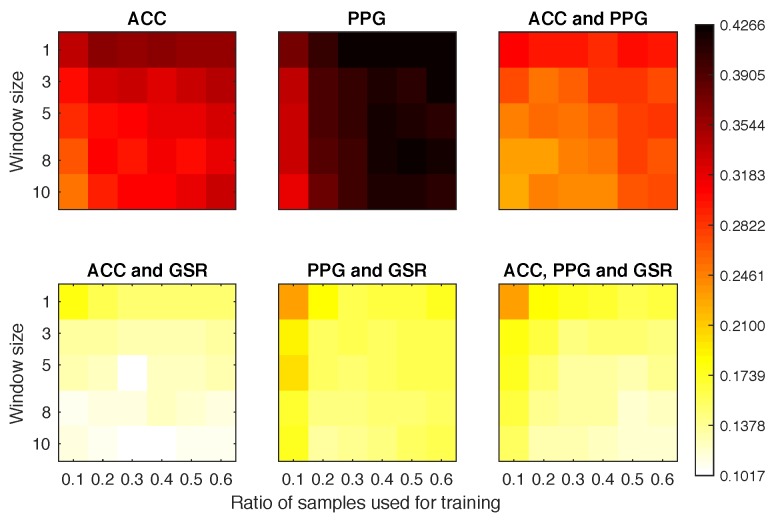
Equal error rate obtained for each experimental configuration in Scenario 1b.

Figure 7 plots the EER obtained for Scenario 1b experiments. It is clear that the signals ACC and PPG offered very poor performance on their own. When combined with the GSR they achieved a much better overall performance than any other combination. In fact, the configuration using 10% of samples as training set and 3 windows for classification obtained an EER of 0.14.

#### 4.4.3. Scenario 2

**Figure 8:**
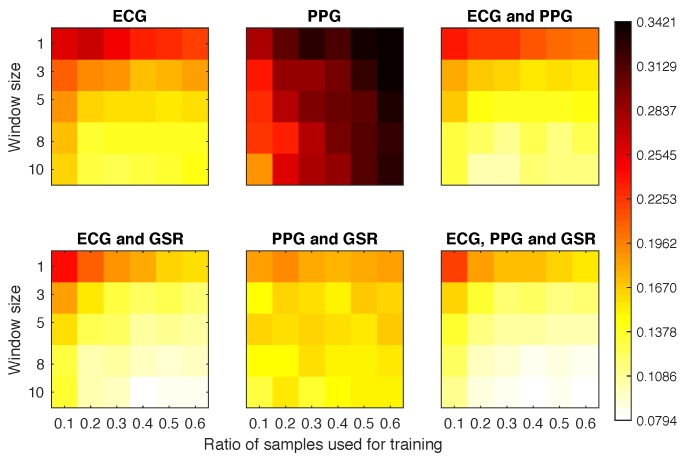
Equal error rate obtained for each experimental configuration in Scenario 2.

Again, the PPG offered the worst overall results (Figure 8). However, even with the classifier being tested against samples from a different activity, we still reached EER ≤0.13 for most of the configurations where the PPG was not involved and the window size was above 5. The best overall classifier was obtained with ECG and GSR, obtaining EER = 0.079 with only 40% of the sample data and 10 windows for classification.

#### 4.4.4. Scenario 3

**Figure 9:**
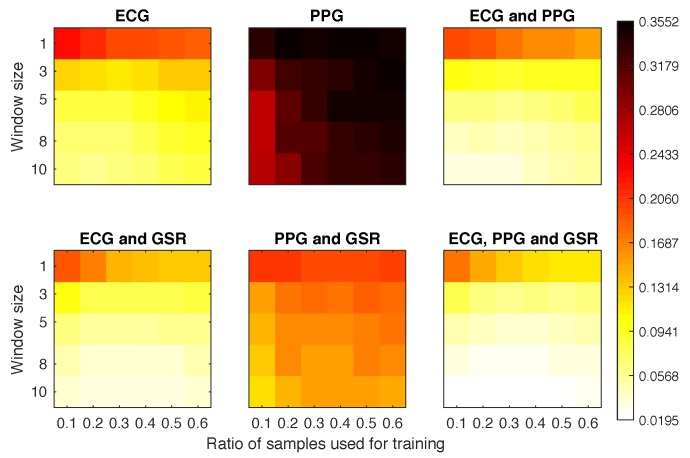
Equal error rate obtained for each experimental configuration in Scenario 3.

The results displayed in Figure 9 show an improvement over the previous scenarios. Once we started using three windows for the classification we obtained EERs below 0.08 when combining ECG with any of the other signals. For most of these configurations, the EER results were homogeneous, having small differences between configurations.The poor results of the PPG signal suggest that this physiological signal should not be used alone, but as a complementary identifier.

### 4.5. Comparing Scenarios

Table 4 shows the best configuration obtained for each scenario (AUC and EER). Our best results were obtained in Scenario 3 in which two activities were considered in training and testing. It also worth noting that, although it is not the most realistic setting, using samples taken from only one activity in both training and testing is standard practice used in most ECG-based biometric research. This is because the vast majority of public datasets exist for medical purposes (e.g., PhysioBank Databases [45]) and participants are typically resting during the data acquisition.

**Figure 10:**
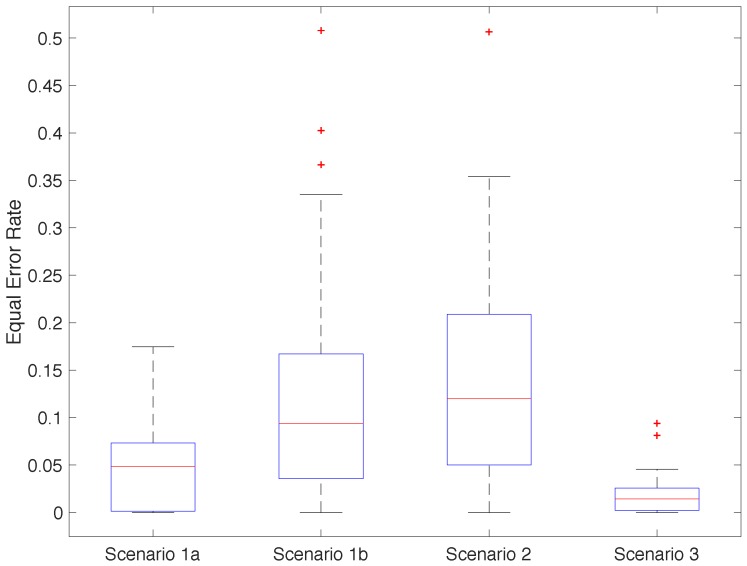
Distribution of the EER obtained for each participant and scenario (averaging repetitions).

Figure 10 shows the EER distribution among participants for each scenario in their best configuration. Participants in Scenario 3 had a relatively uniform behaviour, with two outliers—participants 5 and 16. Scenario 1a showed a similar behaviour to Scenario 3, but with a wider distribution of values. Scenarios 1b and 2 showed very heterogeneous EER with the same outliers as in 3. We suspect that the variability of classifier quality in these two scenarios came from the noise while walking in Scenario 1b and the absence of samples from activity 3 in Scenario 2.

**Figure 11:**
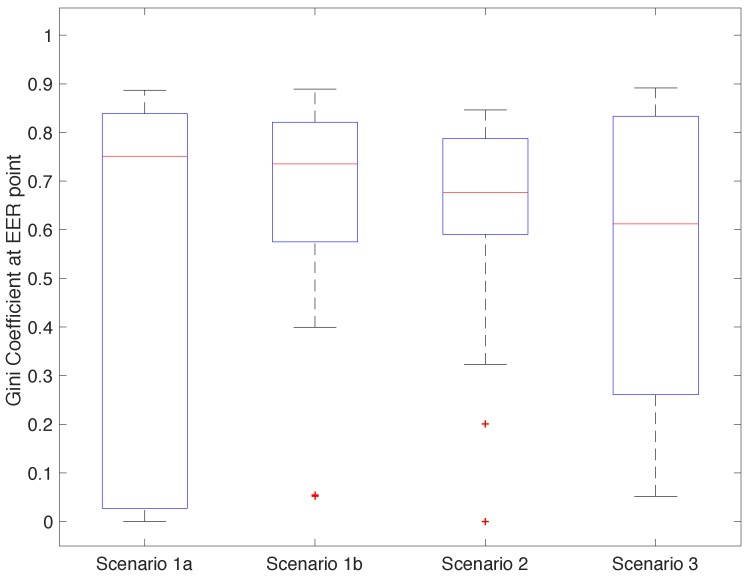
Distribution of the Gini coefficients when the classifier is operating at EER.

Figure 11 shows the distribution of Gini coefficients among participants for the best configuration in each scenario (operating at the EER) [20]. A Gini coefficient closer to 1 means that the same participant would be able to systematically authenticate as the target user, defeating the purpose of the system. A Gini coefficient closer to 0 means that errors were distributed equally among the participants, reducing the risk of systematic bypass. All scenarios had a high but similar median. This implies that many of the errors generated by the classifiers came from the same users. This is especially worrying for Scenarios 1b and 2, as they had a relatively high EER and Gini coefficients. Participants who can bypass the system in these cases can do it consistently. For Scenarios 1a and 3, many participants had relatively low Gini coefficients. In conjunction with the low EER, this implies that in most cases, it is difficult to find a participant that can impersonate another participant consistently.

**Figure 12:**
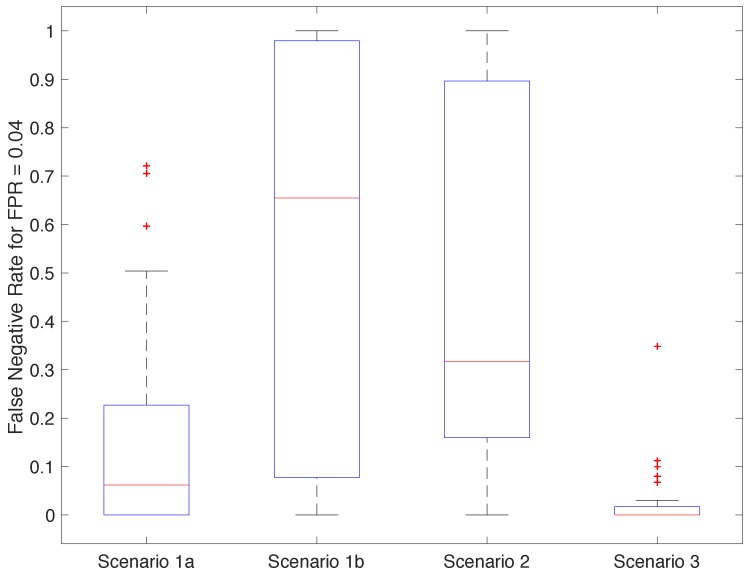
Distribution of the false negative rate (FNR) obtained for each participant and scenario with false positive rate (FPR) = 0.04.

To investigate how we can reduce the possibility of this happening even further, we placed the point of operation of our best configurations to obtain a false positive rate equal to 0.04. Figure 12 shows the distribution of the false negative rates across the participants, for each scenario, under this specific setting. Most of the classifiers from Scenarios 1b and 2 became unusable under this configuration. Scenario 1a offered generally good results with the exceptions of the outliers, which this time included participants 5 and 17. Most classifiers in Scenario 3 had a false negative rate of 0, with a few exceptions, including again participant 17, close to 0.35.

## 5. Discussion

The worst results were obtained in Scenarios 1b and 2, with the accelerometer (EER = 0.1071) and when training and testing on different participant states (EER = 0.0794). The variability introduced by the five-minute walk increased the average EER by 0.06, matching the results in [15]. Da Silva et al. observed an increase in the EER of 0.08 when the ECG measures were taken four months apart. These results lead us to believe that using a single user state for training is not the best strategy for ECG-based systems.

When training with samples from different activities (Scenario 3), we obtained classifiers with EER = 0.07 with just 60 s of data (20% of samples for training) by using three windows (6 s) and ECG, PPG, and GSR. Increasing the training time reduced the EER to 0.06 while increasing the window size to 10 (20 s) for testing improved the EER to 0.02.

Participants 5, 16, and 17 always generated bad classifiers. We suspect this is because the prototype was not properly adjusted during the data collection process, resulting in readings with many artifacts.

Table 5 compares our results with previous related works. Cornelius et al. developed a biometric verification system using the GSR signal [17] that achieved 0.85 accuracy and 0.127 EER over small populations of participants (5 to 8). Other works performing verification achieved an EER of 0.11 and 0.05 over 78 and 21 participants when using ECG and ACC, respectively [16,18]. Our configurations outperformed or matched all of the other works, with the exception of [19], which works in identification mode. Considering that, as previously noted, this comparison is not entirely fair, we believe that the combination of the three biosignals (ECG, PPG, and GSR) can significantly improve the quality of the biometric system.

With regard to the dataset used, there are some limitations. Generally, biometrics systems must comply with four requirements: (1) collectability; (2) universality; (3) unicity; and (4) permanence [46]. Our results clearly guarantee requirements 1–3. Regarding permanence, in the used dataset, the signals were acquired only once on a given day. Previous works show how physiological signals such as ECG and PPG—which are the most fruitful signals in our system—suffer slight variations after a long period of time [15]. Despite this, this represents a limitation of our dataset, and an in-depth analysis of the permanence property (e.g., cross-day and long-term analysis) is recommended in a future study.

## 6. Conclusions

In this work, we analysed the feasibility of using low-cost wearable sensors to build a multi-modal biometric system to perform user verification. Our results showed that the implementation of such systems in a realistic setting is feasible, but several challenges must be considered. First, low-cost sensors that are being worn continuously by the user are subject to movement-generated noise that can reduce the quality of the captured signal. This could be mitigated by a proper fit of the device that limits its movement. Second, signals like ECG, PPG, and GSR vary over time because of changes in the user state or ageing. To avoid false negatives under these circumstances, a biometric system should allow the addition of new samples over time to keep the biometric system under acceptable metrics (as explored in our third scenario).

Our biometric system does not include any protection against device theft, replay, or spoofing attacks. We acknowledge that for some systems like ECG there are methods to replay a previously captured signal [23]. However, we believe that a multi-modal system that uses ECG, PPG, and GSR practically increases the complexity of such attacks. There are no known ways to replay a PPG signal directly to its sensor, and it is very challenging to correctly manipulate the GSR. Independent of this, we showed how important it is to verify the metrics of the classifiers obtained for each of the subjects—as shown in Figure 11, the Gini coefficient helps to identify whether a particular user could cheat the system systematically.

To complete and generalise the dataset, as future work, users may be recorded on different days (preferably repeating it over a long period of time) for the proposed set of scenarios. In turn, for each scenario, subjects may be under different situations (relaxation, stress, fear, etc.) and places (indoor or outdoor). The set of scenarios could also be made larger by expanding the number of activities (e.g., running, cycling, or driving) that users execute during the data acquisition. In addition, another aspect to analyse in depth is related to when and how often the user credentials are validated. In the proposed system, regular time intervals were used. As a future work, credentials could also be evaluated continuously. This is the gap between a classical identification system and a continuous identification system. Data stream mining techniques may be used for this purpose. These types of systems are promising since they can cope with the concept of drift (slight variations over time), as these commonly occur in biosignals.

## Figures and Tables

**Table 1:** Features extracted per each 2-s window. ACC: accelerometer; *F*: Fourier transform; GSR: galvanic skin response; *H*: Walsh–Hadamard transform.

Signals	Features
$ECG_{j}$	$F_{ECG}\left[0,1..31\right]$, $H_{ECG}\left[0,1..63\right]$
$PPG_{j}$	$F_{PPG}\left[0,1..31\right]$, $H_{PPG}\left[0,1..63\right]$
$ACC_{j}$	$F_{ACC}\left[0,1..31\right]$, $H_{ACC}\left[0,1..63\right]$
$GSR_{j}$	$\bar{GSR_{j}}$, $\sigma (GSR_{j})$, $max(GSR_{j})$, $min(GSR_{j})$

**Table 2:** Data captured for each participant.

Ord.	Act.	Dur.	Signals	# Samples
1	Sitting	5 min	ECG, PPG, GSR	25×130
2	Walking	5 min	PPG, ACC, GSR	25×130
3	Sitting	3 min	ECG, PPG, GSR	25×70

**Table 3:** Activities and signals for each scenario.

Scenario	Train	Test	Signals
1a	Activity 1	Activity 1	ECG, PPG, GSR
1b	Activity 2	Activity 2	ACC, PPG, GSR
2	Activity 1	Activity 3	ECG, PPG, GSR
3	Activities 1 and 3	Activities 1 and 3	ECG, PPG, GSR

**Table 4:** Parameters and best average metrics for each scenario. AUC: area under the curve.

Scenario	Features	Train	Window Size	AUC	EER
1a	ECG, PPG, GSR	60%	10	0.982	0.053
2b	ACC and GSR	30%	10	0.937	0.107
2	ECG and GSR	40%	10	0.966	0.079
3	ECG, PPG, GSR	30%	10	0.996	0.019

**Table 5:** Comparison with other proposals using ECG, ACC, PPG, or GSR signals.

Work	Signals	Mode	EER	Subjects
**S1a**	ECG, PPG, GSR	Verif.	0.05	25
**S1b**	ECG, PPG, GSR	Verif.	0.11	25
**S2**	ECG, PPG, GSR	Verif.	0.08	25
**S3**	ECG, PPG, GSR	Verif.	0.02	25
[17]	GSR	Verif.	0.127	8
[19]	ECG	Ident.	0.01	30
[18]	ECG	Verif.	0.11	78
[16]	ACC	Verif.	0.05	21

**Figure A1:**
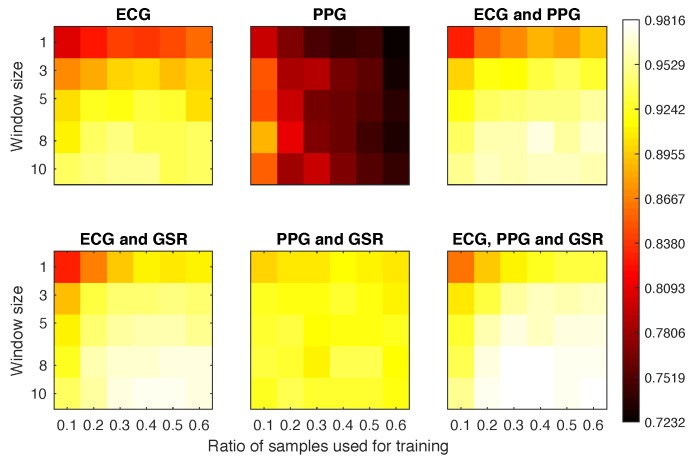
Area under the curve (AUC) obtained for each experimental configuration (averaging participants and repetitions) in Scenario 1a.

**Figure A2:**
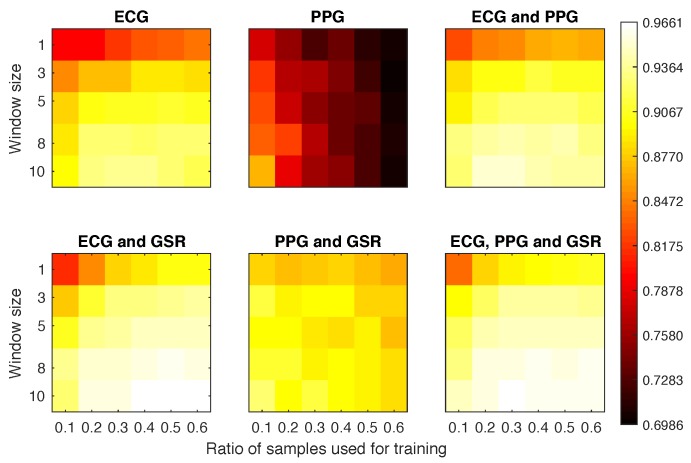
Area under the curve (AUC) obtained for each experimental configuration (averaging participants and repetitions) in Scenario 2.

**Figure A3:**
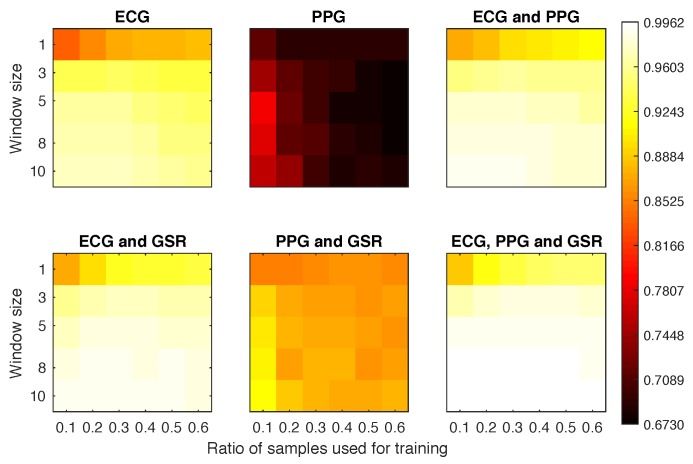
Area under the curve (AUC) obtained for each experimental configuration (averaging participants and repetitions) in Scenario 3.
